# Broadband transfer of binary images via optically long wire media

**DOI:** 10.1515/nanoph-2022-0538

**Published:** 2023-01-16

**Authors:** Dmytro Vovchuk, Mykola Khobzei, Mykhailo Apostoliuk, Vladyslav Tkach, Constantin Simovski

**Affiliations:** Department of Radio Engineering and Information Security, Yuriy Fedkovych Chernivtsi National University, Chernivtsi, Ukraine; Department of Electronics and Nanoengineering, Aalto University, Espoo, Finland

**Keywords:** binary imaging, broadband transfer, resolution, wire media

## Abstract

In the paper the binary mechanism of the long-distance image transfer in a wire-medium (WM) endoscope is suggested and studied. We have shown that a discrete image formed by a set of point sources TM-polarized with respect to the WM can be transferred from the input to the output of the endoscope in a very broad frequency band. The underlying physics is the formation of local channels by a group of four adjacent wires. It allows the transfer of the near field beyond the Fabry–Perot resonances at which the known canalization mechanism offers the image. Both numerical simulations and experimental measurements confirm the deeply subwavelength resolution on the output WM interface. The binary imaging was studied until the frequencies at which the endoscope length exceeds 5*λ*. The transfer is possible in the entire investigated frequency range (from 1 up to 4 GHz) except for the frequencies where the Fabry–Perot resonance are not enough suppressed.

## 1 Introduction

Wire media (WM) considered as multi-wire transmission lines represent a tool for power transfer in narrow and broad frequency ranges. It is possible for low radio frequencies, for microwaves and even mm waves – up to far-infrared band [1–4]. Also, WM layers are suitable for subwavelength imaging. It was first shown in [5] and since 2005 this direction has been developed in many works (e.g. in [6–11]). In these works, the near-field image was transferred at the frequencies of the WM layer Fabry–Perot resonances. However, the operation at the resonances restricts the functionality due to binding to geometrical sizes of WM. It is important to note that the transfer of the near-field image of a complex object and the transfer of the power for a source are tightly related problems. Both types of transfer are implemented by the packages of the WM sample eigenmodes. In a lossless WM layer (infinitely extended across the wires) these eigenmodes are TEM waves. For a finite-width WM sample such as WM endoscope these are TM (quasi-TEM) waves [10, 12], [13], [14], [15].

The broadband effect of the power transfer in the WM layer (not endoscope) by TM-waves was first theoretically shown in [15] and since that time has been confirmed in several works. For example, at microwave frequencies it was experimentally proven in [12, 13] for a finite-width sample whose length *L* of the wires was comparable with the transverse sizes of the sample slightly inserted into two hollow waveguides distanced by nearly the same gap *L* without an electric contact of the wires and the waveguides. The power transfer in that system implied the broadband conversion of TE polarized plane waves forming the main eigenmode of a waveguide into quasi-TEM modes of the WM sample. This phenomenon seemingly paved the way to WM endoscopes dedicated for subwavelength imaging in a broad frequency band and WM spectroscopes. However, broadband imaging in a WM endoscope was not yet achieved neither at radio nor at optical frequencies.

In works such as [8, 16], [17], [18], [19], [20] pixel pictures of a WM endoscope at the frequencies of Fabry–Perot resonances were shown for a variety of frequency ranges: MHz, GHz, THz, mid and near infrared frequencies. Moreover, in [14] a harmful effect was revealed – on the surface of long WM samples called WM endoscopes optical vortices arise at some frequencies and prevent the power transfer. This effect does not interfere with broadband power transfer, as it operates within a set of very narrow frequency ranges. However, this obstructs the WM endoscope from bending, since bending shifts these frequencies, and one or more of them may turn in the operating range of the endoscope.

In this work, we do not consider bending of the elastic endoscopes. We concentrate on the lacuna in the theory of straight WM endoscopes. On the one hand, the broadband power transfer was proven for them, on the other – binary (pixel-to-pixel) imaging i.e. imaging with subwavelength resolution was achieved in WM samples solely at the Fabry–Perot frequencies. However, the power transfer and the image transfer are related to each other. Though the subwavelength image is formed by the near fields of the object, it is transferred to the output of the endoscope by propagating waves to which the near field is converted at the WM interface. Therefore, if the broadband power transfer is possible, the broadband mechanism of the image transfer should also exist. Here, this mechanism is revealed and shown to offer the broadband binary imaging with subwavelength resolution in the optically long WM endoscope. We demonstrate its feasibility at radio frequencies for which we managed to confirm our hypothesis and calculations experimentally.

## 2 Conceptual idea and its possible implications

The key difference of our imaging mechanism from the resonant mechanism (canalization) is as follows – in order to be resolved spatially the point sources should be located nearly in the middles of the squares formed by the groups of four adjacent wires. Under this condition the transfer of the whole image is performed by an array of parallel guiding channels into which the WM endoscope effectively splits.

**Figure 1:**
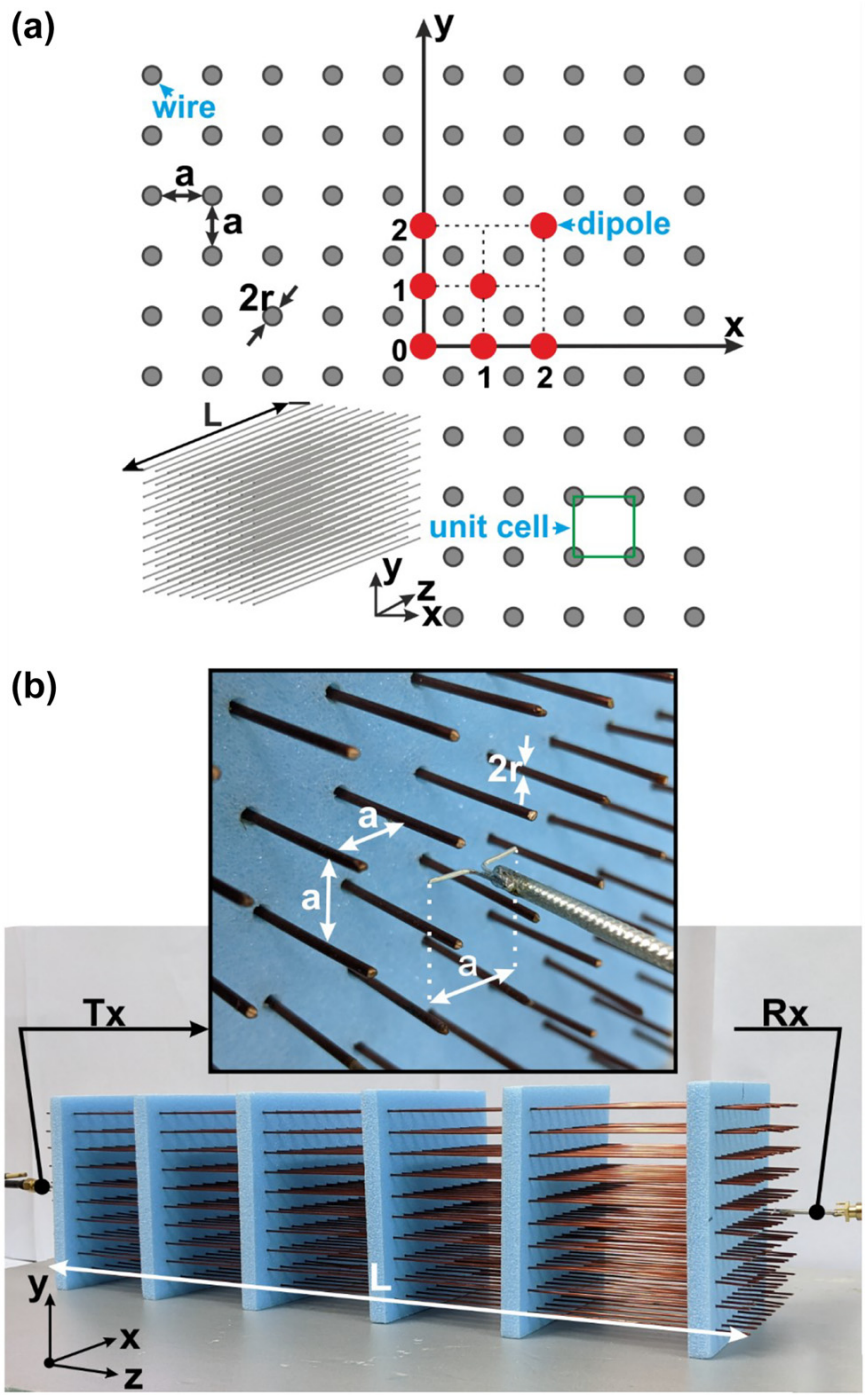
Transverse cross section of the WM sample (*L* – wire length, *a* – lattice period, *r* – wire radius) and the positions of the receiving dipoles (red circles) in the output (*xy*) plane (*z* = 0) (a). The single transmitting source can be located in the center of any unit cell in the plane *z* = *L*. The experimental sample of WM with *L* = 400 mm, *a* = 10 mm and *r* = 0.5 mm and the Tx/Rx dipole antennas inserted contactless inside the WM with arm length *a *(b).

We represent the WM as an array of unit cells of length *L* having the square cross section *a*-by-*a.* Unit cells are formed by four quarters of four adjacent wires, as shown in Figure 1a. We have assumed that beyond the resonances of the WM sample these unit cells can operate as independent waveguides enabling the transfer of power by a quasi-TEM wave from point sources located in the middles of some unit cell to the output interface of the WM sample. If so, the binary image transfer should be broadband since in our four-wire transmission lines the inductance and capacitance per unit length are mutually balanced, in other words the propagation factor and the characteristic impedance are frequency independent.

The canalization mechanism of subwavelength imaging does not work for a WM slab beyond the Fabry–Perot resonances due to the strong reflection of the radiation from the WM interfaces (both input and output). Every point source, located in front of the end of any single wire, excites the oscillating charge at this end which in turn results in the excitation of the whole interface by every point source. Therefore, the near fields spread over the interfaces of a WM slab, and the pixel-to-pixel imaging becomes impossible. Only at the Fabry–Perot resonances this near-field spread does not occur because the WM interfaces are matched to free space, and the charges on the ends of the wires turn out to be small. However, if the TM-polarized point source is located in the middle of the unit cell, it quasi-statically interacts with four surrounding wires and excites them as if it was placed in the middle of a cable. The interaction of the source with its own effective channel presumably dominates over its interaction with all other wires, and the near field of the source is not spread over the input interface. If so, the mutual interaction of the sources across the WM sample is also damped and Fabry–Perot resonances are not needed for transferring the near fields of the sources along the endoscope.

One may think that our type of imaging is impractical because possible locations of the point sources are restricted by centers of the effective channels. However, we believe that this imaging is useful though it is not imaging in the usual sense but the transfer of near-field images. Really, our WM endoscope is a digital telegraph transferring the image of the matrix of elements in which some elements are bright and others are dark. The information about the location of the bright and dark elements is transmitted over large distances without an electric contact with both matrices – that of transmitting (Tx) and that of receiving (Rx) elements. It may be used, e.g. in the IR sensing.

In the IR optics the Tx matrix can be that of quantum light-emitting diodes having the submicron transverse sizes and emitting broadband radiation in the mid-infrared range [21] and the Rx matrix can be that of charge-coupled devices (CCD), that is also a broadband array and may be engineered for operation in the mid-IR range. The WM endoscope may link these matrices having the internal period as small as *a* = 1.12 μm determined by the recently achieved granularity of the CCD matrix [22]. Thus, the broadband image formed in the mid-IR range such as *λ* = 3–10 μm by quantum LEDs can be transferred by the WM endoscope over optically large distance with a deeply subwavelength resolution. Then the electronic signal from the CCD matrix can be used for detection of the broadband IR image. Here submicron LEDs should operate as sensors [23].

Our device can be also applied in the visible light subwavelength imaging if we replace the CCD matrix by photosensitive nanorods [23]. Then we have to reduce the period of the wire medium from 1.12 μm to, say, 150–200 nm (experimentally achieved lateral sizes of quantum dots). Next, this mechanism can be also used in the THz imaging, where the broadband subwavelength image can be obtained in an electro-optical crystal (see e.g. in [23]) whose surface is stacked to the input interface of the WM endoscope. The WM endoscope transfers the image to THz spectroscopic devices. THz endoscopes are diverging and enable significant magnification of the image [18]. This magnification does not depend on the signal band, and we obtain a tool for the analysis of the image extracted from an electro-optical crystal.

We also bear in mind the possibility of a magnified image in the diverging WM endoscope engineered for the visible range. It seems a promising alternative to usual tips of a scanning near-field optical microscope (SNOM). In this case, we may implement the regime of photon emission when the light travels from the cantilever towards the object. Our device is reciprocal and only the object points located in front of the centers of our effective channels will be illuminated. Knowing the coordinates of these points we may obtain their spectroscopic image in the scattered light. Implementing the tip of a SNOM as a tapered array of metal (silver) nanowires we will drastically enlarge the aperture of the tip compared to a usual SNOM. This modification will significantly accelerate the object scanning.

However, the first stage of our study is the imaging in a WM endoscope of parallel wires. In the present paper we only aim to prove the subwavelength imaging at the distances of the order of several wavelengths from the object.

## 3 Study of the spatial resolution at radio frequencies

To investigate the resolution of an imaging device means to determine the minimal distance between the sources which are distinguished at the output interface in accordance with the Rayleigh criterion. However, in the present structure, where the granularity is fixed as *a*, we only need compatibility between *a* and the Rayleigh criterion. If the coupling coefficient of the unit cell, where the Tx dipole is located to the neighboring source, is lower than 0.7 at a given frequency it means that the resolution at this frequency is *a.* If the coupling is higher, we have to check the coupling with unit cells whose axes are distanced by 2*a* from the source, etc. We also do not need to study what is *M* in the spatial resolution of our device postulated as *R* = *Ma*, as only Tx dipole is enough for it. Therefore, we locate our Tx dipole at the centers of different unit cells moving it in the input interface from the initial point (*x*, *y*, *z*) = (0, 0, *L*-5). Recall that the input plane is *z* = 0 and *L* = 400 mm. However, this movement of the source is needed only to determine the influence of the sample lateral surface. In the proximity of the corner of our WM sample the impact of two lateral sides of our WM sample (parallel to *x* and parallel to *y*) can be different. Then our M splits into two integers. Accordingly, we locate a number (*P* = 5) Rx dipoles centered in *N* unit cells (see also in Figure 1a) with coordinates (*x*, *y*, *z*) = (*na*, *ma*, Δ*z*), where *n* and *m* = 0:1:2, and Δ*z* = 395 mm. A usual electric dipole antenna was selected as a source of EM signals for the simulations in CST Microwave Studio and then for experimental measurements.

The length of our dipole sources (9 mm) was small enough to locate them in our unit cell and the resonance frequency of these dipoles (∼15 GHz) was far from the operational frequency range in order to imitate the weak EM wave radiation. It also allows neglecting by cross-talk in the case of dipole array (S_21_-parameters of the located in adjacent cells dipoles are less than −50 dB). The WM constructive parameters *L* = 400 mm, *r* = 0.5 mm and *a* = 10 mm were picked up for the investigations in GHz frequency range from 1 up to 4 GHz which corresponds to *L* = ∼1.3*λ* … 5.3*λ*. The frequencies less than 1 GHz were not considered because of that frequency region corresponds subwavelength power transfer while the condition of the long-distance imaging is the goal of study here that was respected for the pointed-out frequency range.

To estimate the resolution, it was necessary to investigate contrast between direct (*S*_21_^00^-parameters) and cross-transmission (*S*_21_^*xy*^-parameters) via the ratio *T*_00_^xy^ = *S*_21_^xy^/*S*_21_^00^. During the measurements the Tx dipole was fixed at the input interface with coordinates (*x*, *y*) = (0, 0) whereas the Rx dipole was moving step-by-step along the horizontal (*x*), vertical (*y*) and diagonal (*x* and *y* together) axes. Therefore, the indices in *T*_00_^*xy*^ mean the following: ‘00’ are the coordinates of the Tx dipole fixed in the origin, and ‘*xy*’ are the coordinates of Rx dipole shifted by one-two unit cells along the *x*, *y* or both *xy*-axes.

**Figure 2:**
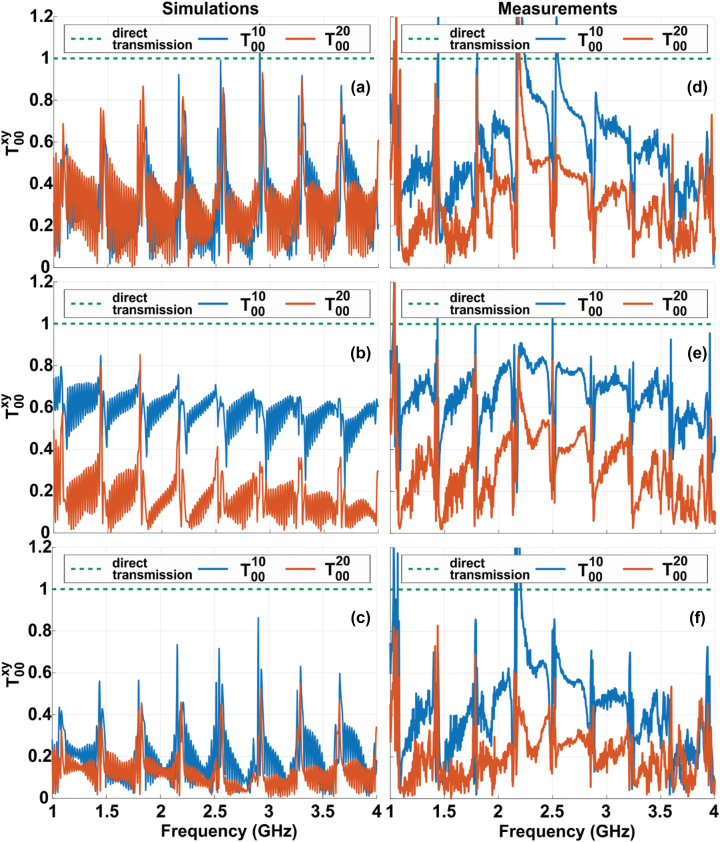
Simulation (1st column) and measurements (2nd column) results for the cross-transmission *T*_00_^*xy*^ in the case of (a) *x* = 1*a*, 2*a* and *y* = 0*a*; (b) *x* = 0*a* and *y* = 1*a*, 2*a* and (c) together *x* and *y* are equal to 1*a*, 2*a*. Except the specific location of the excited effective channel in (a) and (c), the signal transfer in the neighboring effective channels is much lower than the signal transfer in the own effective channel of the source (at least beyond the bands of the Fabry–Perot resonances). The green dashed line is equal to ‘1’ and corresponds to direct transmission.

We aimed to receive by Rx dipole the maximal signal power for direct transmission and as less as possible for the cross one. The ratios *T*_00_^*xy*^ will show us how much direct transmission prevails other cross-transmissions. Therefore, one can see from Figure 2a–c (the first column) that the resolution is equal to one lattice period value *a* due to the *T*_00_^*xy*^ << 1 in almost whole investigated frequency range except the very narrow frequency regions at non-suppressed Fabry–Perot resonances. At these exceptional frequency regions one can see *T*_00_^*xy*^ > 1 where the reason is resonant behavior of the wires of WM and strong coupling between them. It causes a transferring possibility via the neighboring cell as well. Thus, frequencies must be avoided, while the suggested transfer approach is being used. This effect will be considered in detail below when the pixel recognition.

For the measurements the WM sample was manufactured with the same geometrical and structural parameters as the simulated model (Figure 1b). The material of the wires is copper. The blue dielectric holder in Figure 1b is transparent for EM waves in the operational frequency range. Two electrical dipoles correspond to the simulated one.

Measurements, whose results are shown in Figure 2d–f (the second column), confirm the assumed mechanism of the signal transfer in the broad frequency range 1–4 GHz along parallel channels of the WM sample. The experiment fits the simulations and shows the possibility to distinguish the Tx dipoles located onto the input interface of the sample with deeply subwavelength gap *a* by an array of Rx dipoles arranged with the period *a* on the output interface. However, here we can see that the ratios *T*_00_^10^, *T*_00_^01^ and *T*_00_^11^ are closer to unity in comparison with the simulation results especially for the diagonal case. The Rayleigh criterion is not respected for the case when the distances between the point sources is equal *a* – the resolution is not as fine. If the distance between the Tx dipoles is 2*a* the resolution will correspond *R* = *S*/(4*a* ^2^) = *nma* ^2^/(4*a* ^2^) = *nm*/4, where *S* = *nma* ^2^ – the WM interface square, where *n* and *m* are the amount of wires along the horizontal and vertical axes of WM, respectively.

## 4 Binary imaging in the broad frequency range

**Figure 3:**
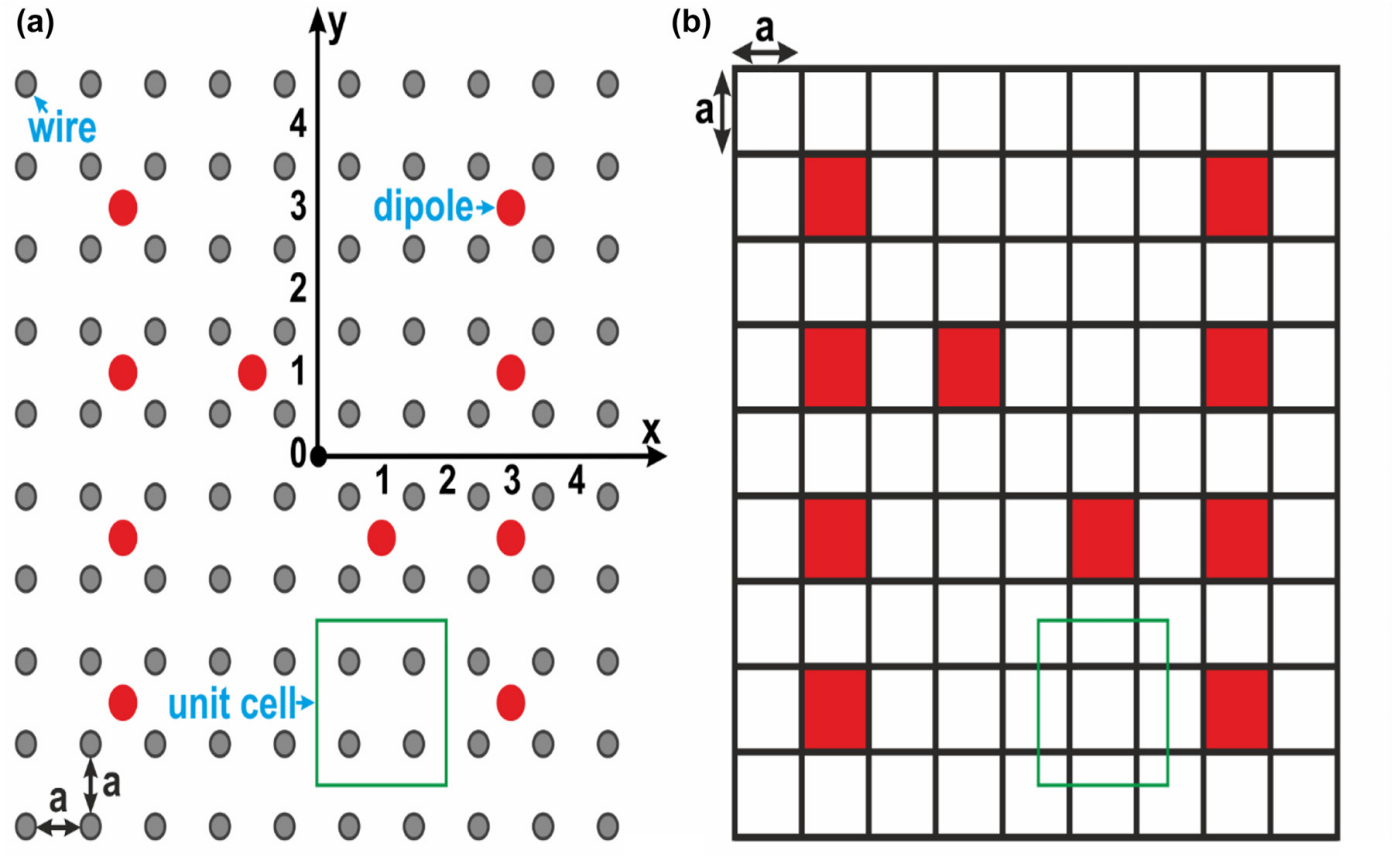
(a) The arrangement of sources forming letter *N* as a transmitting picture onto the input WM interface and (b) the expected view of a detected picture at the output interface of WM.

In accordance with the aforementioned results, which indirectly point out the spatial resolution equal to 2*a*, the object was formed by dipoles shown in Figure 3a by red circles. The distance between the dipoles is 2*a* and the object’ shape is letter *N* that includes all possible interactions between adjacent sources: horizontal, vertical and diagonal. Thus, one pixel of our image is 2*a*-by-2*a* as it is shown at the inset of Figure 3a. In the ideal case we expect to detect at the output interface of our endoscope the maximal power values at the positions corresponding to the positions of Tx sources as shown in Figure 3b and zero values at other positions of Rx antennas.

**Figure 4:**
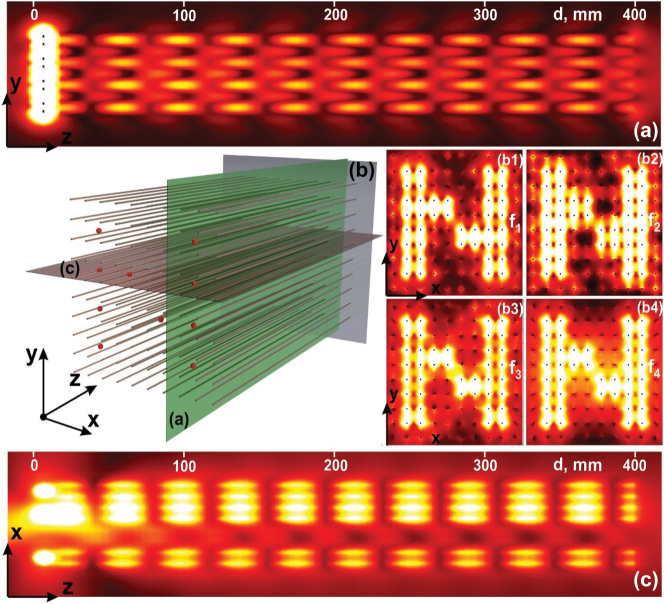
Color maps of the electric intensity distribution in the *yz*-plane for *x* = 30 mm (a), in the output interface (the *xy*-plane for *z* = 395 mm) at the frequency *f*_1_ = 2 GHz (b1), *f*_2_ = 3 GHz (b2), *f*_3_ = 3.5 GHz (b3) and *f*_4_ = 3.85 GHz (b4) and in the *xz*-plane for *y* = 10 mm (c). The red spots depict the sources.

The three-dimensional model of the WM endoscope is shown in Figure 4 together with the color maps of electric field intensity. The positions of the sources are depicted as red spots at the input interface. Three planes colored by magenta (horizontal), also referred to as c, green (referred to as a) and grey (referred as b) are planes for which we show the electric field color map. These planes were selected with the coordinates: in the *yz*-plane at *x* = 30 mm for the green one (Figure 4a); in the *xy*-plane at *z* = 395 mm for the grey one (Figure 4b); and in the *xz*-plane at *y* = 10 mm for the red one (Figure 4c).

The map in Figure 4a shows the power flow from the four different dipoles along the four effective channels with minor coupling between them. This is confirmed by the map in Figure 4c – top view on the electric intensity distribution confirms our qualitative assumption that the effective channels are naturally decoupled.

Finally, the color map in Figure 4b shows us how the input intensity distribution (red spots in Figure 4c) is transferred to the output interface of the endoscope. Here, we represent the output intensity distribution for the frequency *f*_1_ = 2 GHz on the panel b1, *f*_2_ = 3 GHz – b2, *f*_3_ = 3.5 GHz – b3 and *f*_4_ = 3.85 GHz – b4. As can be seen, the letter *N* is reproduced very well at both frequencies.

## 5 Experimental verification

**Figure 5:**
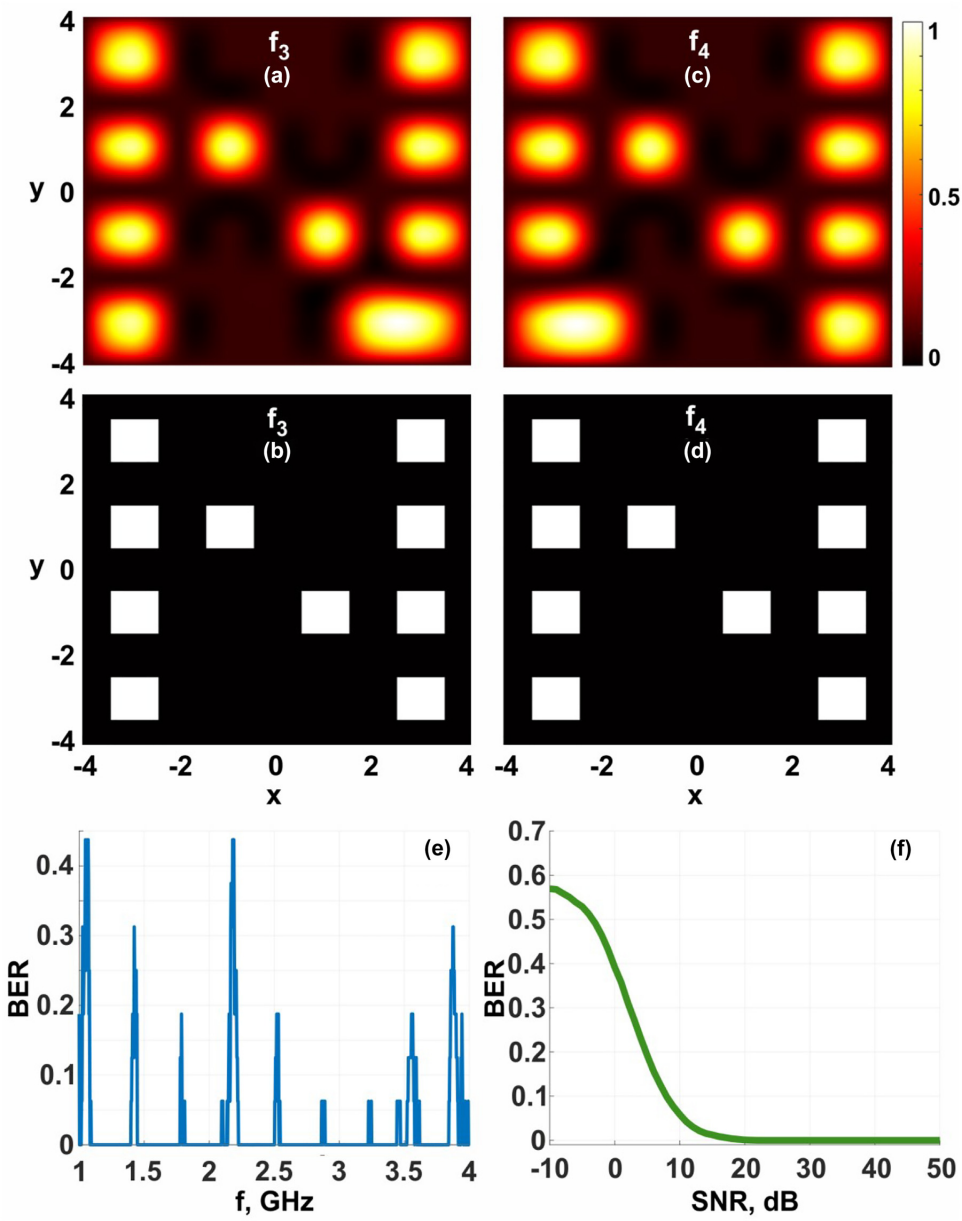
The experimentally obtained E-field distributions for the transfer of the binary image shaped as *N* at two frequencies (a for *f*_3_ and c for *f*_4_) and their binary representation after the thresholding procedure (b and d, respectively). The frequency dependence values of BER (e) and BER versus SNR at the frequency *f*_3_ (f). AWGN was applied for the experimentally measured data.

For the experimental confirmation of our idea, we built the sample pictured in Figure 1b with the sources placed as shown in Figure 3a. Instead of creating an N-shaped array of Tx dipoles we used one Tx dipole and one Rx dipole that we shifted between the unit cells recording the transmitted power for each mutual location. This way we numerically synthesized the image following to the superposition principle. Our experimental results are depicted in Figure 5 as the normalized E-field amplitude distributions for frequencies *f*_3_ = 3.5 GHz and *f*_4_ = 3.85 GHz. The higher frequencies were picked out because of the purpose of the optically long WM demonstration of functionality. For better visualization the bicubic spline and shading interpolations were applied to the image data. One can see that the recognition of the letter N is highly possible in the investigated range (Figure 5a and c).

For a binary representation of the scanned field distributions of image we applied a simple approach for digital signal post-processing based on a threshold to convert image to binary. In order to automate the processing, the threshold was chosen for each picture using Otsu’s method [24]. The method defines a global threshold value through the minimization of intraclass of the black and white pixels after the thresholding. The threshold is defined from the range [0, 1], therefore the field distributions in Figure 5b and d were normalized to their maxima.

We applied the binary conversion to each separate cell at the output interface which corresponds to the coordinates of possible locations of the sources at the input interface. It means we have analyzed all the positions *x* = −3:2:3 and *y* = −3:2:3 separately, but applied the same threshold to all of them. For example, the scanned field from the source with coordinates (*x*, *y*) = (−3, −3) gives the maxima at (*x*, *y*) = (−3, −3) and (−3, −2) (Figure 5c). However, we have not considered the maximum at (*x*, *y*) = (−3, −2) during the digitalization due to the previously defined statement about sources arrangement with 2*a* step. As a result, the binary images for the frequencies *f*_3_ and *f*_4_ are shown in Figure 5b and d, respectively. In accordance with the imperfection E-field distribution before the thresholding, depicted in Figure 5a and c, the even the simple digital algorithm is capable of reconstructing the transferred picture.

The frequencies *f*_3_ and *f*_4_ for the presented reconstructed images were picked up between the Fabry–Perot resonances from Figure 2 where *T*_00_^*xy*^ < 1. In the case when *T*_00_^*xy*^ is greater than or close to 1, image recognition is complicated due to the presence of a number of false pixels the reasons for which have been explained earlier. Quantitatively it can be described with calculation of the values of bit error ratio (BER) for the digitized images at all frequencies from the investigated range (Figure 5e). BER means the ratio of the number of incorrectly reconstructed recovered bits (number of errors *n*_er_) to the total number of transferred bits *N* (1). Considering the images in Figure 5b and d, one can see *N* =16 pixels (or bits). It means that the presence of one error gives an error of 0.0625. Therefore, such analysis is usually applied to a much larger number of bits. However, the analysis gives the opportunity the estimate and define the frequencies where the transfer is possible. To analyze and search for erroneous pixels, we created a ‘correct’ matrix of pixels (2) and compared with the one after thresholding. As a result, it is evident that the maxima of BER(*f*) are caused by the Fabry–Perot resonances, while the perfect reconstruction is between them (Figure 5e). Some exceptions can be seen along the frequency axis caused by the presence of a single error, which appears as a large value due to the limited number of pixels.

(1) $$BER=\frac{n_{\text{er}}}{N}$$

(2) $$S_{\text{correct}}=\left(\begin{array}{ccc} x_{11} & \ldots & x_{k1}\\ \vdots & \ddots & \vdots \\ x_{1l} & \ldots & x_{kl} \end{array}\right)$$

where the ‘on’ pixels are highlighted as *x*_*kl*_ = 1 and ‘off’ ones – *x*_*kl*_ = 0 for the matrix dimension *k*-by-*l* = 9-by-9.

Another important characteristic is the ability to recognize an image under noise impact. The additive white Gaussian noise (AWGN) was used as a noise imprint. We applied AWGN to the measured data utilizing the standard MatLab function. BER was estimated versus signal-to-noise ratio (SNR) for the perfectly reconstructed image (at the frequency *f*_3_). SNR means how much the signal power exceeds the noise power and, in our study, it was in the range from −10 to 50 dB (Figure 5f).

However, it is noteworthy that this advantage does not apply to the ‘clear’ signal, but to the measured signal which already includes some additive noise that could be present in the laboratory (3). Considering that the noise power distribution is a random process (the ratio means the averaged values of the measured power field distribution and AWGN power), we calculated BER(SNR) for 100 samples and depicted the averaged plot in Figure 5e. As a result, a near-zero BER can be seen for an SNR value below 20 dB, which is comparable (but not quite due to the restricted numbers of pixels in our study) with 8- and 16-PSK modulation characteristics.

(3) $$SNR=\frac{P_{\text{signal}}+P_{\text{lab}.noise}}{P_{\text{AWGN}}}$$

where *P*_signal_ is the power of the signal in each single pixel and *P*_lab.noise_ is the power of possible noise in the laboratory during the experiment and this noise was supposedly added to the signal; as well as *P*_AWGN_ is the power of additive white Gaussian noise.

## 6 Conclusions and discussions

In the paper we have suggested and studied a new mechanism of the long-distance transfer of subwavelength images in wire medium endoscopes. Unlike previously studied mechanism known under the name of canalization, our mechanism is broadband. It allows one to obtain the non-resonant images of the points located at the center of the squares formed by four adjacent wires. The spatial resolution is equal 2*a,* where *a* is the WM period. In terms of wavelength, it is equal 0.07*λ* − 0.27*λ* for the range 1–4 GHz. The distance (up to *L* = 5.3*λ* at 4 GHz) at which this resolution was obtained is restricted only by the available room sizes and the operational frequency range of the equipment. This distance can be increased without compromising the superresolution. It allows the maximal number of pixels in the image *R* = *nm*/4, where the EM endoscope comprises *n-*by-*m* wires. This binary image is not as rich as that in the case of canalization [17], where *R* = *nm*, however, this drawback is compensated by the possibility to have a spectroscopic image due to very broad band of the endoscope operation.

In this paper, we have discussed possible applications of such WM endoscopes – from THz to the visible range, including as yet unexplored divergent variants of such endoscopes. Note that, in addition to imaging, the property of the nearly independent signal transfer in adjacent effective channels excited by different sources can be used to replace microwave and mm-wave waveguide arrays with a very simple counterpart, namely WM sample.

Moreover, the waveguides in microwave and mm-wave arrays are quite narrowband, whereas our WM endoscope can be used for the transfer of many poly-harmonic and broadband signals in the different parts of the same sample. At the same time, while we considered in the paper the transfer from the active EM wave sources, the detection of passive weakly radiative sources is possible. It can find an application in sub-THz and THz endoscopy and spectroscopy taking into account the manufacturing technologies shown in [1, 25], [26], [27], [28]. Based on this, we believe that the presented results will find an application at different frequency ranges. As a perspective usage of endoscopes [14, 16, 18, 29] can be developed for the digital case with the further medical purpose [30]; material science for surface spectroscopy [31, 32], security devices for detection of explosive or narcotic substances but with the possibility to penetrate instead of remote detection [33, 34]; and many others. At the same time, we suppose that the perspective in the optical band is declared in [14] due to the advantage over the optical fiber because of the bending angle up to 180° with the radius less than wavelength without any changes in transmission characteristics. However, the losses are still must be decreased that is an issue for the future work. The EM wave propagation in the sub-GHz range and microwaves (and not only [29, 35, 36]) can be developed for the multi-channel waveguides up to at least 4 GHz as was show in this paper or even broader [12, 16] and to increase the resolution of WM-based metasurfaces [37–39]. However, the high losses are an open problem for WM waveguides [14] and it is a one of the important tasks for the future work.

We believe that our novel concept, our results and the identified features of the WM endoscope will open up new opportunities for useful applications of wire metamaterials.
